# Carmustine as a Supplementary Therapeutic Option for Glioblastoma: A Systematic Review and Meta-Analysis

**DOI:** 10.3389/fneur.2020.01036

**Published:** 2020-09-17

**Authors:** Zhi-Ze Xiao, Ze-Fen Wang, Tian Lan, Wen-Hong Huang, Yu-Hang Zhao, Chao Ma, Zhi-Qiang Li

**Affiliations:** ^1^Department of Neurosurgery, Zhongnan Hospital of Wuhan University, Wuhan, China; ^2^Department of Physiology, School of Basic Medical Sciences, Wuhan University, Wuhan, China; ^3^Laboratory of Neuro-Oncology, Zhongnan Hospital of Wuhan University, Wuhan, China

**Keywords:** glioblastoma, carmustine (BCNU), overall survival (OS), meta-analysis, chemotherapy

## Abstract

**Background:** Glioblastoma (GBM) is the most aggressive type of primary malignant brain tumor. Carmustine is used by intravenous injection or local implantation in the resection cavity for gliomas, including GBMs. However, the therapeutic potential of carmustine is not well-recognized. This analysis aimed to evaluate the survival benefits of carmustine in glioma patients, especially those with GBM.

**Methods:** Randomized controlled trials (RCTs) and cohort studies regarding carmustine for glioma treatment were searched in PubMed, the Cochrane Library, and Embase from January 1979 to March 2020. Quality assessment was conducted with Jadad and Newcastle–Ottawa scales (NOS). Statistical analysis was conducted by the Revman 5.3 software.

**Results: Twenty-two** eligible RCTs and cohort studies involving 5,821 glioma patients were included. Overall, glioma patients receiving carmustine as an adjuvant therapy had better progression-free survival [PFS; hazard ratio (HR) = 0.85, 95% CI = 0.77–0.94, *P* = 0.002] and overall survival (OS; HR = 0.85, 95% CI = 0.79–0.92, *P* < 0.0001) than those without carmustine treatment. Subgroup analysis showed that the OS benefit was observed in GBM (HR = 0.84, 95% CI = 0.78–0.91, *P* < 0.00001) but not in anaplastic glioma patients (HR = 1.20, 95% CI = 0.70–2.07, *P* = 0.50). Additionally, both newly diagnosed and recurrent GBM patients who received carmustine treatment showed better OS (HR = 0.86, 95% CI = 0.79–0.95, *P* = 0.002; HR = 0.77, 95% CI = 0.67–0.89, *P* = 0.0002, respectively). Both carmustine implantation in resection cavity and intravenous administration significantly prolonged OS (HR = 0.84, 95% CI = 0.78–0.92, *P* < 0.0001; HR = 0.86, 95% CI = 0.75–0.99, *P* = 0.04, respectively). Moreover, GBM patients receiving a combined carmustine and temozolomide (TMZ) therapy had longer OS than those receiving TMZ alone (HR = 0.78, 95% CI = 0.63–0.97, *P* = 0.03).

**Conclusion:** Carmustine implantation in resection cavity provides survival benefit for GBM patients, and it may be a promising supplement to standard therapeutic protocol by offering a bridge between surgical resection and onset of TMZ therapy.

## Introduction

Glioma is the most common primary malignant central nervous system tumor. Glioblastoma (GBM), World Health Organization (WHO) grade 4 glioma subtype, is one of the most deadly malignant tumors with an estimated incidence of 5.26 per 100,000 population or 17,000 new diagnosed patients per year (1). GBMs are present at the median age of 64 years, but can occur at any age, including in children (2). The standard care for newly diagnosed GBM is maximal safe surgical resection, followed by radiochemotherapy with the alkylating agent, temozolomide (TMZ). However, the tumor inevitably recurs, and standardized strategies for the treatment of recurrent glioma are lacking. The evidences of a favorable outcome regarding re-resection and re-irradiation are still poor (3). Therefore, systemic chemotherapy has been explored as a prospective option for glioma.

The nitrosourea derivative, carmustine {BCNU, [1,3-bis (2-chloroethyl)-1-nitrosourea]}, another alkylating agent, has also been used both at the initial diagnosis of glioma and at tumor recurrence, either by intravenous administration or by wafer implantation. Biodegradable wafers impregnated with carmustine (Gliadel® wafer) and implanted in the surgical bed on the walls of the resection cavity (4) were developed initially by Brem et al. to avoid the toxicity associated with systemic administration of carmustine (5). Carmustine wafer implantation in GBM patients undergoing surgical resection is also thought to provide a therapeutic bridge during the period between surgical resection and onset of radiotherapy (6). Previous research assessing the effectiveness of carmustine wafer has found a significant increase in overall survival (OS) by 2–4 months in newly diagnosed GBM patients (3). Although the efficacy of carmustine administration is established in seminal trials, its safety remains controversial. The common side effects of carmustine-based chemotherapy include nausea/vomiting and hematotoxicity with a delayed nadir after 4–6 weeks, and the most dreaded side effect, pulmonary fibrosis. For carmustine wafer implantation, its impact on post-operative infections, quality of life, and feasibility of adjuvant oncological treatments has also been debatable (6). The experimental data of carmustine administration are rather controversial, and there is no general agreement about adverse events. A systematic understanding on whether carmustine wafer contributes to survival in glioma patients is still lacking.

This study aimed to assess whether carmustine treatment is beneficial for GBM and other gliomas, and whether the addition of carmustine therapy as a supplement to STUPP protocol could provide more survival benefits for GBM patients.

## Methods

### Search Strategy

A literature search was performed by two independent reviewers across three databases including PubMed, Embase, and the Cochrane Library. Randomized controlled trials (RCTs) and cohorts involving carmustine for glioma treatment were retrieved by searching databases from January 1979 to March 2020. For the search, the following string of terms was used: (astrocytoma OR oligodendroglioma OR oligodendroglial OR glioma OR glioblastoma) AND [carmustine OR BCNU OR N,N′-bis (2-chloroethyl)-N-nitrosourea OR 1,3-Bis (2-chloroethyl)-1-nitrosourea OR FIVB (fluorouracil, imidazole carboxamide dimethyl triazeno, vincristine, and bis-chloroethyl nitrosourea) OR BCNU OR Nitrumon OR carmustine wafers OR Gliade]. Reviews and references of included studies were also checked to avoid omission of relevant publications. The studies were restricted to human beings.

### Selection Criteria

RCTs and cohort researches meeting the following criteria were considered eligible: (1) RCTs regarding carmustine treatment in glioma patients irrespective of blinding methods and publishing language, and cohort studies regarding carmustine treatment in glioma patients; (2) studies with comparison of therapeutic regimen with or without carmustine; and (3) studies containing the hazard ratios (HRs) for OS, progression-free survival (PFS) or survival curves, or details from computed data presented.

RCTs and cohort studies meeting the following criteria were excluded in this meta-analysis: (1) duplicate reports or studies lacking adequate original data and (2) studies in which patients suffered from other primary tumors, trauma, or severe infections.

### Data Extraction and Quality Assessment

Two researchers read the titles and abstracts of the identified literature, excluding the irrelevant ones, reviews, and pharmacological experiments. Full texts of the possibly pertinent RCTs and cohort studies were checked further to determine whether they fulfilled the inclusion criteria. The reference sections of the retrieved articles were also screened. Case reports, animal experiments, editorials, and letters were excluded. If the eligible studies provided both univariate and multivariate analyses, hazard ratios (HRs) calculated by multivariate analysis were preferred as these values had higher precision for interpreting confounding factors in the Cox regression model. The two researchers conducted their quality evaluations independently. If there were disagreements, decisions were made following discussions or further inquiry by a third researcher.

RCTs using the Jadad scale (ranging from 0 to 5) were considered to be of high quality (7). The Newcastle–Ottawa scale (NOS) was used to assess the quality of the included cohort studies (8). There were three main aspects including study selection (0–4 points), comparability (0–2 points), and study outcomes (0–3 points). Scores of 6 points or more were deemed to be of relatively high quality.

### Statistical Analysis

All calculations and graphs were made using the Revman 5.3 software (The Nordic Cochrane Center, The Cochrane Collaboration, 2014). Time-to-event data (e.g., OS) were analyzed using the HRs. Chi-squared test was used to evaluate heterogeneity among studies, and *I*^2^ was used to measure the magnitude of heterogeneity (9). Results with *P* ≥ 1 and *I*^2^ ≤ 50% indicated a lack of significant heterogeneity; in such cases, the Mantel–Haenszel fixed-effects model was used for meta-analysis. Subgroup analysis was performed to evaluate the possible source of heterogeneity and to further study the preliminary results. If there was no obvious heterogeneity, the fixed model was used to estimate the pooled HR (10). Otherwise, the random-effects model was used (11). Sensitivity analysis was performed to evaluate the risk of bias of studies. Publication bias was assessed by visually examining the funnel plots (12). A trim-and-fill method was applied to estimate asymmetry in the funnel plot (13).

## Results

### Characteristics of the Studies

**Figure 1:**
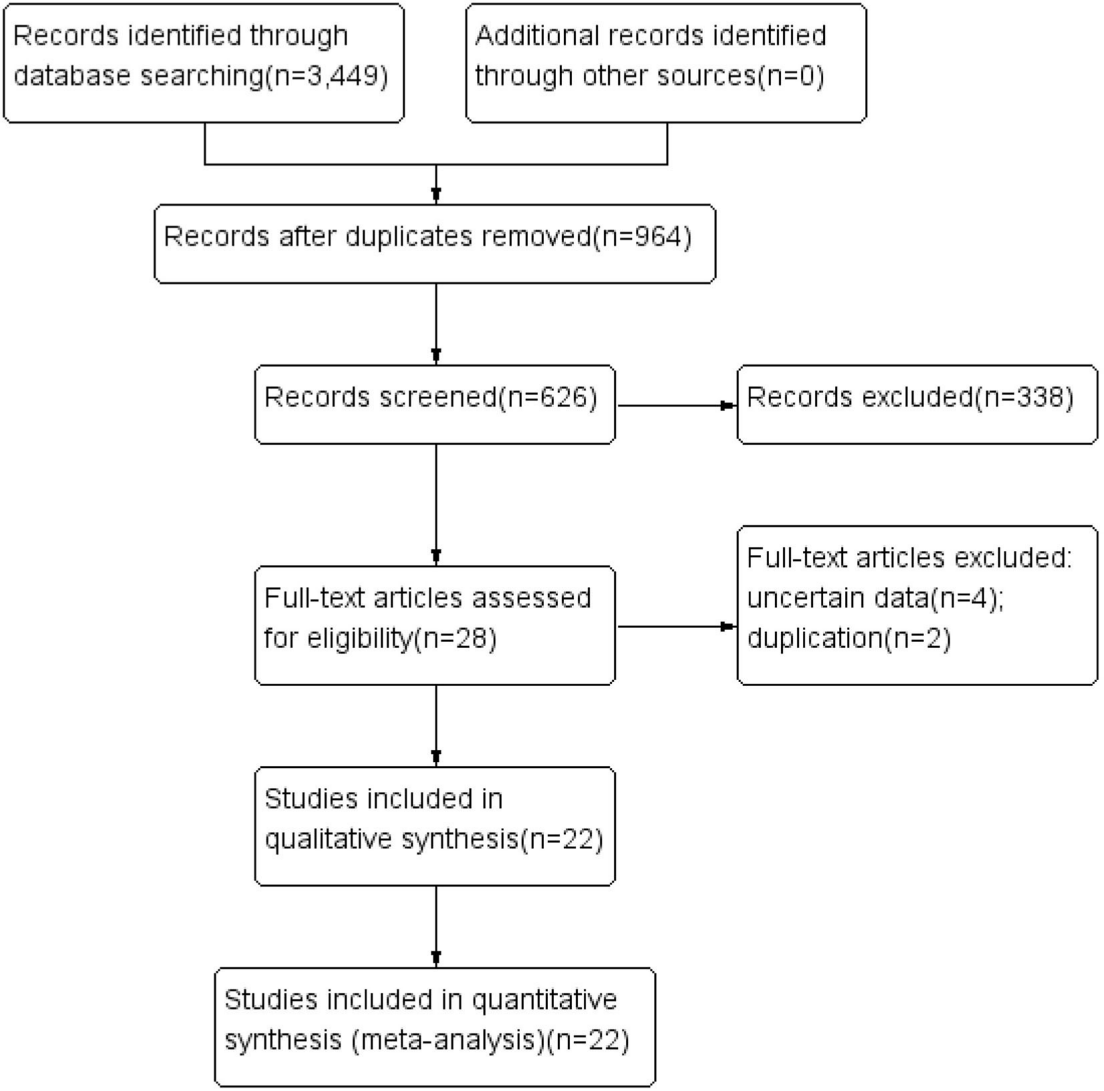
Flow chart of the literature selection.

A flow chart of literature selection is shown in Figure 1. A total of 3,449 articles were screened via a primary search of the literature database. After reading the titles and abstracts to remove irrelevant items, and reading the full texts to eliminate those that did not meet the inclusion criteria, we obtained 28 research reports that evaluated the OS of glioma patients treated with carmustine. Finally, four uncertain and two duplicate studies were excluded, and a total of 22 studies (9 RCTs and 13 cohort studies) involving 5,821 glioma patients were included in this meta-analysis [6, 14–34]. The characteristics of the included studies are shown in Table 1. In the included literature, 19 studies were carried out in GBM patients, and two studies were in anaplastic glioma patients, and one study was in patients with gliomatosis. Six RCTs used a placebo-controlled, double-blind, randomized method (14–19). In the other three RCTs, implantation of carmustine was not randomized and was decided according to clinical conditions (6, 20, 21). The comparability of one cohort study is obscure (22), while that of others is relatively distinct (23–34). The results of the methodological quality assessment using NOS and Jadad scale are also shown in Table 1.

**Table 1:** Characteristics of included studies.

**Study**	**Year**	**District**	**Patients with/without carmustine**	**Adjuvant therapy (with/without carmustine)**	**Study design**	**Medication method**	**Grade of glioma**	**Quality of studies(score)**
Affronti et al. (23)	2009	Occident	36/49	RT and concurrent TMZ plus rotational chemotherapy.	Cohort	Resection cavity	GBM	NOS (9)
Autran et al. (24)	2019	France	15/7	TMZ/Other treatment	Cohort	Intravenous	Gliomatosis	NOS (8)
Brem et al. (14)	1995	USA	72/73	RT and systemic chemotherapy	RCT	Resection cavity	GBM	Jadad (5)
Chaichana et al. (25)	2010	USA	148/192	Adjuvant radiation and chemotherapy	Cohort	Intravenous	GBM	NOS (7)
Chaichana et al. (26)	2014	USA	64/195	RT and TMZ	Cohort	Resection cavity	GBM	NOS (8)
De Bonis et al. (15)	2012	Italy	10/67	Adjuvant therapy with TMZ	RCT	Resection cavity	GBM	Jadad (4)
	2012	Italy	17/71	Adjuvant therapy with TMZ	RCT	Resection cavity	GBM	Jadad (4)
Esquenazi et al. (21)	2017	USA	42/44	Adjuvant radiotherapy and concomitant TMZ therapy	Cohort	Resection cavity	GBM	NOS (9)
	2017	USA	42/44	Adjuvant radiotherapy and concomitant TMZ therapy	Cohort	Resection cavity	GBM	NOS (9)
Jungk et al. (27)	2016	Germany	34/29	RT and TMZ	Cohort	Intravenous	GBM	NOS (8)
Kunwar et al. (28)	2010	Europe	93/183	Adjuvant therapy	RCT	Resection cavity	GBM	Jadad (4)
Loureiro et al. (20)	2015	Israel	33/82	RT and sequencing chemotherapy/ RT concurrent with chemotherapy	Cohort	Intravenous	GBM	NOS (8)
Louvel et al. (29)	2016	France	254/438	Standard combined chemoradiotherapy	Cohort	Intravenous	GBM	NOS (8)
McGirt et al. (30)	2009	USA	—	TMZ administered with radiotherapy	Cohort	Resection cavity	Anaplastic glioma	NOS (7)
Pallud et al. (22)	2015	France	354/433	Chemoradiation standard protocol	RCT	Resection cavity	GBM	Jadad (5)
Pavlov et al. (31)	2015	France	50/33	Stupp regimen	Cohort	Resection cavity	GBM	NOS (7)
Price et al. (32)	2012	UK	94/202	Radiotherapy and Stupp protocol	Cohort	Resection cavity	GBM	NOS (6)
Roux et al. (6)	2017	France	123/217	Standard combined chemoradiotherapy	RCT	Resection cavity	GBM	Jadad (5)
Sage et al. (33)	2018	UK	78/182	Temozolomide-based chemoradiotherapy protocol	Cohort	Resection cavity	GBM	NOS (8)
	2018	UK	76/76	Temozolomide-based chemoradiotherapy protocol	Cohort	Resection cavity	GBM	NOS (8)
Schold et al. (16)	1993	Occident	128/121	Radiotherapy	RCT	Intravenous	Anaplastic glioma	Jadad (3)
Sun et al. (17)	2015	USA	4/204	Radiotherapy and concurrent temozolomide chemotherapy	RCT	Intravenous	GBM	Jadad (4)
	2015	USA	9/199	Radiotherapy and concurrent temozolomide chemotherapy	RCT	Resection cavity	GBM	Jadad (4)
Valtonen et al. (18)	1997	Northern Europe	5/1	Standard radiotherapy	RCT	Resection cavity	GBM	Jadad (4)
Westphal et al. (19)	2003	Europe	101/106	Radiotherapy	RCT	Resection cavity	GBM	Jadad (5)
Zanello et al. (34)	2017	France	194/583	Standard combined radiochemotherapy	Cohort	Resection cavity	GBM	NOS (8)

*GBM, Glioblastoma multiforme; RCT, Randomized controlled trial; NOS, Newcastle-Ottawa Scale*.

### Association Between Carmustine Treatment and Survival of Glioma Patients

**Figure 2:**
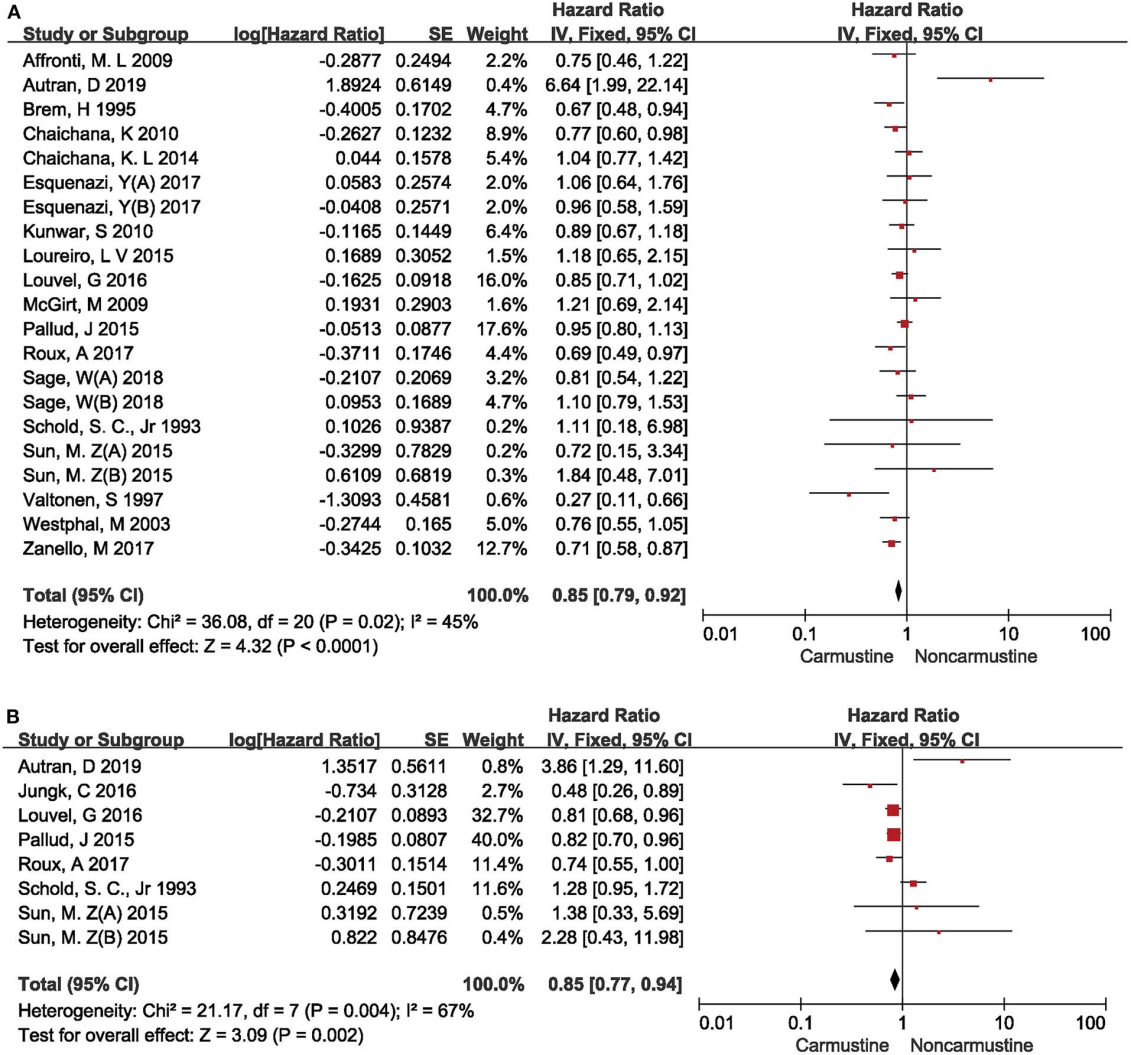
Hazard ratios and 95% confidence intervals of the association between carmustine implantation and overall survival **(A)** and progression-free survival **(B)** of glioma patients. CI, confidence interval; df, degrees of freedom; SE, standard error.

Meta-analysis of the included studies showed that glioma patients treated with carmustine had a better OS (HR = 0.85, 95% CI = 0.79–0.92, *P* < 0.0001, *I*^2^= 45%) (Figure 2A) and PFS (HR = 0.85, 95% CI = 0.77–0.94, *P* = 0.002, *I* = 67%) (Figure 2B) than non-carmustine-treated patients. Since the heterogeneity of survival analysis was high (OS 45% and PFS 67%), the correlation between carmustine administration and survival of glioma patients was further investigated in different subgroups in order to understand the possible source of heterogeneity and to decrease its interference.

**Figure 3:**
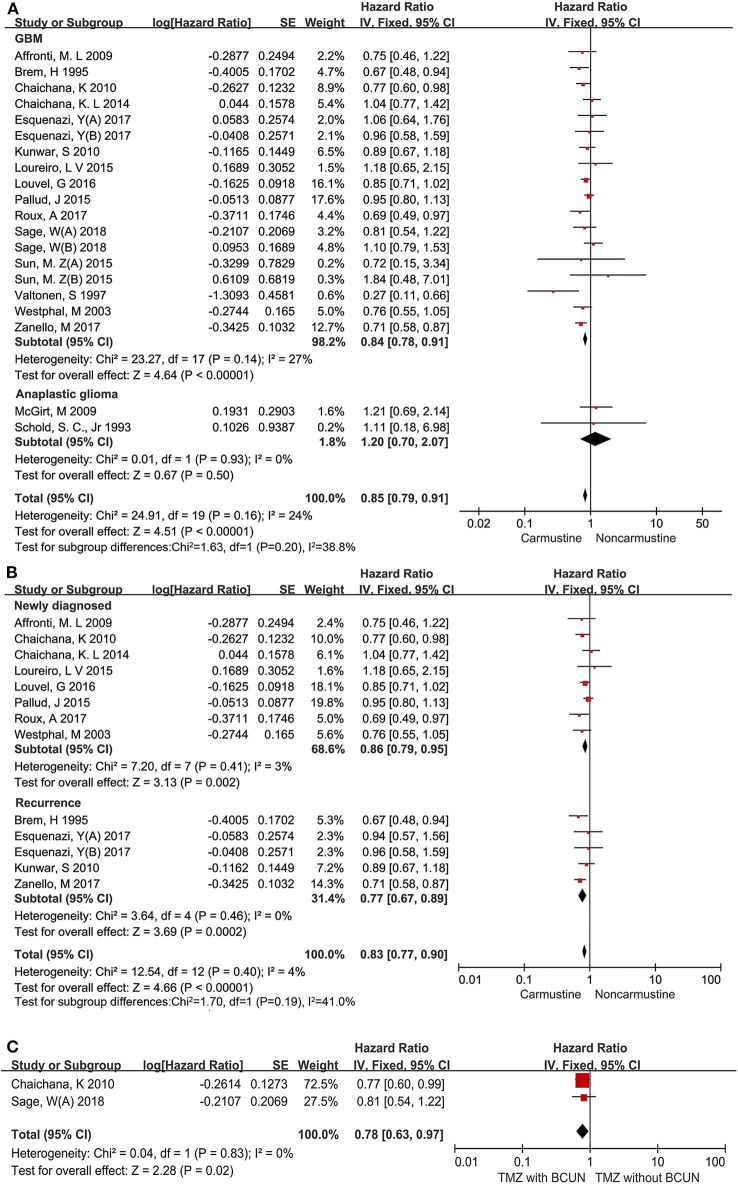
Hazard ratios and 95% confidence intervals of the association between carmustine implantation and overall survival in patients with different pathological grades of glioma **(A)**, in newly diagnosed and recurrent GBM **(B)**, and in patients with TMZ chemotherapy and TMZ plus carmustine chemotherapy **(C)**. CI, confidence interval; df, degrees of freedom; SE, standard error.

It is well-known that the degree of malignancy is the most important factor for the prognosis of glioma patients (35). Therefore, the effect of carmustine on the OS of glioma patients with different pathological grades was first analyzed. Among the included studies, 15 evaluated the OS of GBM patients (6, 14, 17–23, 26, 27, 29, 33, 34), and 2 evaluated the OS of anaplastic glioma patients (16, 30). Carmustine showed a positive benefit on the OS of GBM patients (HR = 0.84, 95% CI = 0.78–0.91, *P* < 0.00001, *I*^2^ = 27%) (Figure 3A). However, there was no OS benefit from carmustine treatment in anaplastic glioma patients (HR = 1.20, 95% CI = 0.70–2.07, *P* = 0.50) (Figure 3A).

The response to therapy is always different between newly diagnosed and recurrent GBM patients (23). Thus, we further evaluated the impact of carmustine on the OS of newly diagnosed and recurrent GBM patients in eight (6, 19, 21–23, 25, 26, 29), and four (14, 20, 27, 29) studies, respectively. This subgroup analysis showed that carmustine treatment contributed favorably to the OS of both newly diagnosed and recurrent GBM patients (newly diagnosed: HR = 0.86, 95% CI = 0.79–0.95, *P* = 0.002, *I*^2^= 3%; recurrent: HR = 0.77, 95% CI = 0.67–0.89, *P* = 0.0002, *I*^2^= 0%) (Figure 3B).

Next, we investigated whether the addition of carmustine therapy as a supplement to STUPP protocol could confer more significant survival benefits for GBM patients. There were only two studies that reported the comparison of survival benefits between patients with TMZ chemotherapy and TMZ plus carmustine chemotherapy (25, 33). The analysis of the two studies showed that GBM patients receiving combined chemotherapy (carmustine plus TMZ) had longer OS than those receiving TMZ alone (HR = 0.78, 95% CI = 0.63–0.97, *P* = 0.02, *I*^2^ = 0%) (Figure 3C).

### Association Between the Administration Route of Carmustine and the Survival of GBM Patients

**Figure 4:**
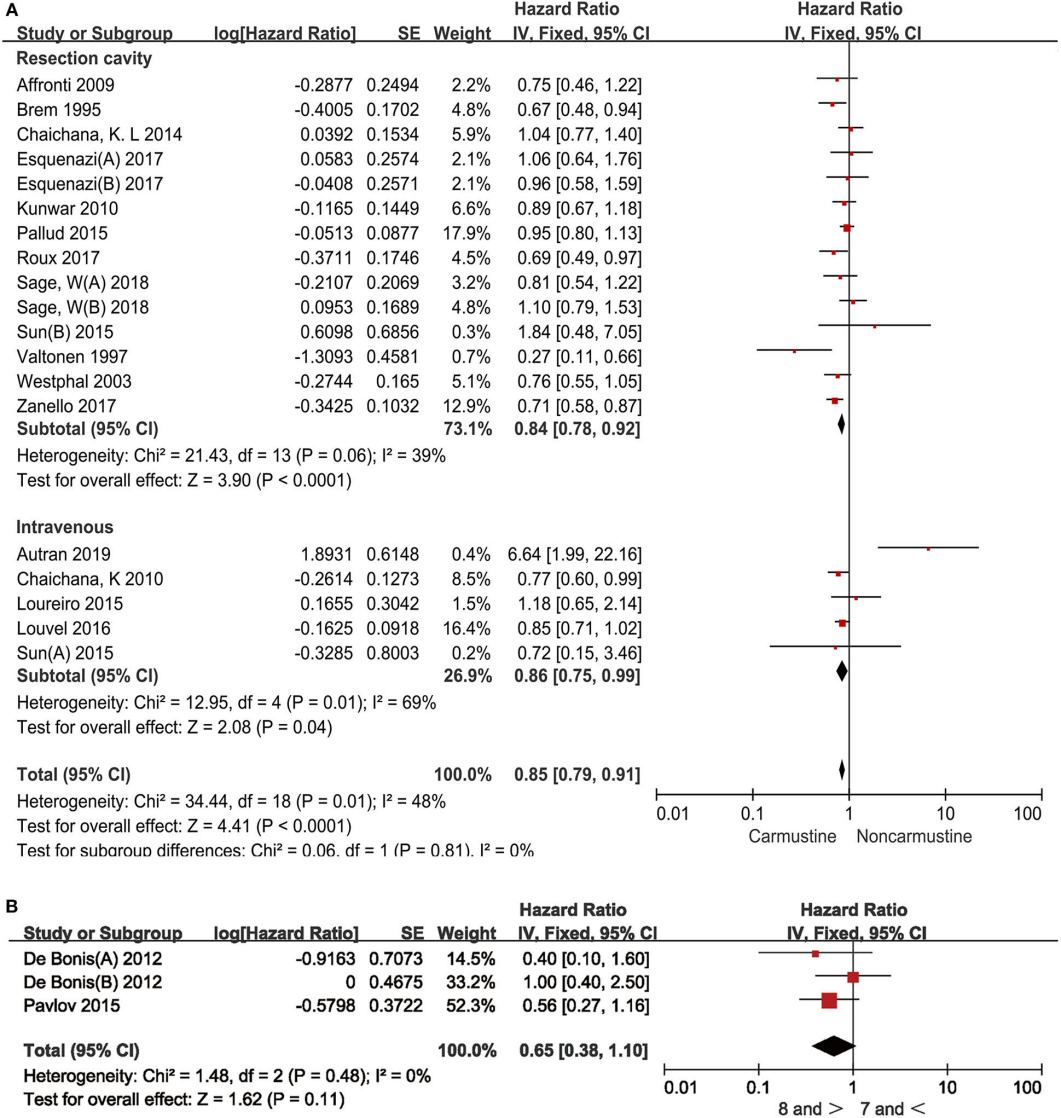
Hazard ratios and 95% confidence intervals of the association between overall survival and administration routes of carmustine **(A)**, and different doses of carmustine wafer **(B)** in GBM patients. CI, confidence interval; df, degrees of freedom; SE, standard error.

Prior to the invention of Gliadel® wafers, carmustine was administered only by intravenous injection. We next examined whether the administration routes had an impact on the survival benefit of adjuvant carmustine chemotherapy in GBM patients. Carmustine was administered by intravenous injection in 6 studies (16, 17, 22, 24, 25, 29) and was implanted into the resection cavity in 12 studies (6, 14, 17–21, 23, 26, 33, 34). Carmustine administrated by both routes prolonged the OS in GBM patients (resection cavity: HR = 0.84, 95% CI = 0.78–0.92, *P* < 0.0001, *I*^2^= 39%; intravenous: HR = 0.86, 95% CI = 0.75–−0.99, *P* = 0.04, *I*^2^= 69%) (Figure 4A).

There is no recommended standard dose of carmustine wafer since the size of the tumor resection cavity varies among patients. Then, we further examined whether the survival benefit of carmustine was associated with the doses used in the studies. The cutoff for the higher dose of carmustine wafer was ≥8 pieces and that for the lower dose was ≤7 pieces. No statistical difference was observed between the two doses (HR = 0.65, 95% CI = 0.38–1.10, *P* = 0.11, *I*^2^= 0%) (Figure 4B).

### Adverse Events

Although there was no severe lethal toxicity, a clinically apparent pulmonary fibrosis caused by intravenous administration of carmustine, with a reported incidence of 0.6–5%, should be considered. As a controversial therapeutic option, carmustine wafer implantation was also reported to result in several side effects. Patients receiving carmustine wafer implantation had higher rates of complications (OR = 1.70, 95% CI = 1.08–2.67, *P* = 0.02, *I*^2^= 15%) than the patients in the non-carmustine group (Supplementary Figure 1). The most common complication with carmustine wafer implantation was post-operative infection. Other adverse events included edema-related intracranial pressure change, healing defect, epileptic seizure, and neurological worsening.

### Publication Bias

A funnel plot was used to measure the publication bias. The funnel plot of the improvement in performance status showed low potential publication bias in the included studies (Supplementary Figure 2).

## Discussion

Radiotherapy combined with concomitant and adjuvant TMZ after maximal safe resection, namely, STUPP protocol, has been widely adopted since 2005 for newly diagnosed GBM (36). Although systemic chemotherapy with TMZ is the most widely used regimen for glioma after tumor resection due to its well-tolerated and favorable clinical outcome, several review researches have reported that carmustine is also beneficial for glioma (37, 38). Previous reviews mainly discussed the survival benefit of carmustine in newly diagnosed GBM. In this study, more subgroup analyses, including glioma grade, newly diagnosed and recurrent GBM, administration routes, and doses, were also performed to comprehensively evaluate the benefits of carmustine in different subpopulation.

Significant OS and PFS benefits of carmustine treatment in glioma patients were observed in this study. The subgroup analysis showed that carmustine chemotherapy was associated with better OS in GBM patients but not in anaplastic glioma patients. More importantly, both newly diagnosed and recurrent GBM patients may benefit from carmustine treatment. It is noted that only two included studies reported the survival outcome in anaplastic glioma patients with or without carmustine chemotherapy. It is also reported that implantation of carmustine resulted in worse OS of patients with leptomeningeal gliomatosis (24). Therefore, it is likely that carmustine may be a reasonable option for patients with GBM but not for those with anaplastic glioma and low-grade glioma. Similar to carmustine, lomustine is always used as a combining chemotherapy agent with procarbazine and vincristine, which is commonly named PCV protocol. PCV regimen was reported to have a similar benefit on OS in patients with anaplastic astrocytoma, and GBM was similar to carmustine (39, 40), while another study showed that PCV was more effective than carmustine in anaplastic astrocytoma patients (41).

Historically, the nitrosourea derivate, carmustine, has been administered intravenously for glioma patients and, lately, has experienced a renaissance in Europe since the invention of biodegradable carmustine wafer. Carmustine administration, along with other adjuvant treatments, is being more frequently utilized because it not only improves OS and PFS but also has been well-tolerated for several years (18, 19). The effectiveness of carmustine wafer was evaluated by RCTs, and the result showed that it could significantly increase OS by 2–4 months in newly diagnosed GBM patients (18, 19). The prognostic advantage of carmustine wafer implantation on OS was also confirmed independent of the extent of resection and the Karnofsky performance score (KPS) (6). This suggested an additional survival benefit of carmustine wafer implantation together with subtotal or total surgical resection at the first surgery of newly diagnosed GBM patients (6). Our study showed that both intravenous administration and carmustine wafer implantation into the resection cavity were associated with better OS in GBM patients. Another published meta-analysis also demonstrated that the median survival time of newly diagnosed high-grade glioma patients who received carmustine wafer treatment was 16 months, and the 1- and 2-year overall survival rates were 67 and 26%, respectively (42). The dose of carmustine wafer is always dependent on the size of the tumor resection cavity, which varies among patients. Therefore, the effect of different doses of carmustine wafer on OS was evaluated, and no statistical difference was observed in our study. In the seven included studies, intravenous carmustine was given 100 mg/m^2^ on day 1 and day 2 or 200 mg/m^2^ on day 1 and repeated every 6–8 weeks. Due to the insufficiency of survival data in the included studies, the correlation between the intravenous dose and the survival outcome was not further examined. In fact, previous studies have demonstrated that longer carmustine exposure duration and lower toxicity could be provided by polymer-based wafer delivery rather than intravenous injection (43).

Although the efficacy of carmustine for GBM is established from our meta-analysis and other seminal clinical trial results, its safety remains a matter of debate. Jungk et al. performed a retrospective analysis on the side effects of carmustine-based chemotherapy in 163 recurrent glioma patients who accepted an intravenous administration of freshly prepared carmustine (28). The results showed that carmustine was well-tolerated with predominantly mild side effects, although 54% of the patients experienced carmustine-related side effects. Interestingly, side effects were not observed equally among tumor grades, with WHO grade IV patients experiencing them least frequently, and WHO grade II patients experiencing them most frequently. The most common adverse event was post-operative infections with carmustine wafer implantation.

As no RCTS or cohort studies comparing the benefits of carmustine alone with TMZ alone were enrolled in our meta-analysis according to the included criteria, we performed the analysis with two published retrospective studies (44, 45) to evaluate the efficacy of carmustine and TMZ. The better efficacy of TMZ alone than carmustine alone was observed in newly diagnosed GBM patients (HR = 0.65, 95% CI = 0.44–0.95, *P* = 0.03, *I*^2^ = 0%) (Supplementary Figure 3). However, carmustine wafer implantation may offer a bridge of “non-therapeutic” period between surgical resection and onset of radiochemotherapy. Our analysis showed that combining carmustine plus TMZ conferred a better OS than TMZ alone, indicating that carmustine wafer may be a supplement to standard STUPP protocol for GBM by offering a bridge of “non-therapeutic” period, allowing continuous chemotherapy. The effectiveness and safety of carmustine as a supplement to STUPP protocol need to be further investigated.

Although TMZ treatment is widely recommended due to its high efficacy, some GBM patients are not sensitive to TMZ therapy, especially those with non-methylated O-6-methylguanine-DNA methyltransferase (*MGMT*) (46). The status of *MGMT* promoter methylation is associated with tumor response to TMZ therapy (46). Our previous study demonstrated the universal predictive value of *MGMT* methylation in newly diagnosed, elderly, and recurrent GBM patients (46). Although the efficacy of carmustine is less than that of TMZ, carmustine may be a reasonable option for GBM patients with non-methylated *MGMT* promoter. Cardona et al. reported that following carmustine/bevacizumab treatment, patients with a combination of an IDH mutation plus MGMT methylation had better OS and PFS than those with only one of these characteristics or those with none (47). Predictive molecular biomarkers for carmustine efficacy need to be further investigated.

### Limitations

The interpretation of the present results should be considered under some limitations. First, the Karnofsky performance score is a crucial factor for deciding glioma treatment choices; however, there are few studies that have calculated HR for KPS. Therefore, we were unable to evaluate the association between KPS and carmustine administration. Second, the relationship between carmustine administration and important prognostic factors, including the extent of tumor resection and tumor molecular features, was not analyzed. We also did not assess the differences in response to BCNU therapy based on sex and ethnicity. Third, we focused on the effectiveness of carmustine alone, while there may be other adjuvant therapies influencing the results as well.

## Conclusion

Carmustine implantation in resection cavity provides survival benefit for GBM patients, and it may be a promising supplement to standard therapeutic protocol by offering a bridge between surgical resection and onset of TMZ therapy.

## Author Contributions

Z-ZX and Z-FW collected the data, performed the statistical analysis, and drafted the report. Y-HZ participated in data collection analysis. TL, W-HH, and CM participated in the interpretation of the data and critically reviewed the manuscript. Z-QL designed the study and contributed to the interpretation and discussion of the results. All authors read and approved the final manuscript. All authors contributed to the article and approved the submitted version.

## Conflict of Interest

The authors declare that the research was conducted in the absence of any commercial or financial relationships that could be construed as a potential conflict of interest.

## Abbreviations

GBM: glioblastoma
Gliadel® wafer: carmustine
BCUN: N,N′-bis(2-chloroethyl)-N-nitrosourea
TMZ: temozolomide
RCTs: randomized controlled trials
NOS: Newcastle–Ottawa scales
PFS: progression-free survival
OS: overall survival
KPS: Karnofsky performance score.
