# Supplementary material for: Carmustine as a Supplementary Therapeutic Option for Glioblastoma: A Systematic Review and Meta-Analysis
Source: Front Neurol. 2020 Sep 17;11:1036. doi: 10.3389/fneur.2020.01036 (PMC7527463; doi:10.3389/fneur.2020.01036)

Supplementary Fig. 1 Hazard ratios and 95% confidence intervals of the occurrence of adverse events in GBM patients with carmustine wafer administration. CI: confidence interval, df: degrees of freedom, SE: standard error

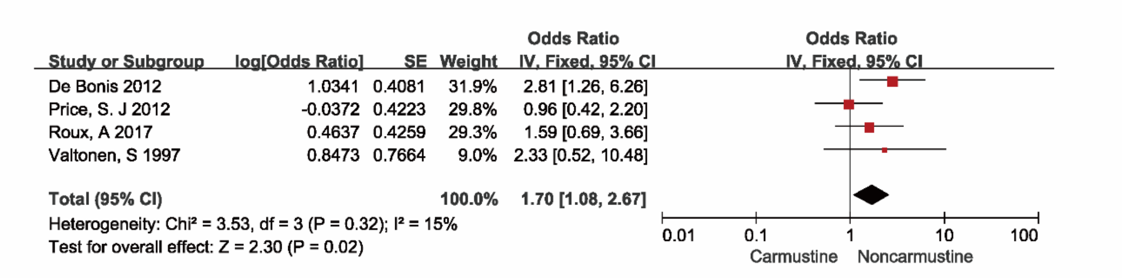

Study or Subgroup	log[Odds Ratio]	SE	Weight	Odds Ratio IV, Fixed, 95% CI
De Bonis 2012	1.0341	0.4081	31.9%	2.81 [1.26, 6.26]
Price, S. J 2012	-0.0372	0.4223	29.8%	0.96 [0.42, 2.20]
Roux, A 2017	0.4637	0.4259	29.3%	1.59 [0.69, 3.66]
Valtonen, S 1997	0.8473	0.7664	9.0%	2.33 [0.52, 10.48]
**Total (95% CI)**			**100.0%**	**1.70 [1.08, 2.67]**

Heterogeneity: Chi² = 3.53, df = 3 (P = 0.32); I² = 15%
Test for overall effect: Z = 2.30 (P = 0.02)

Supplementary Fig. 2 Funnel plot of publication bias: Each dot represents individual studies. SE: standard error

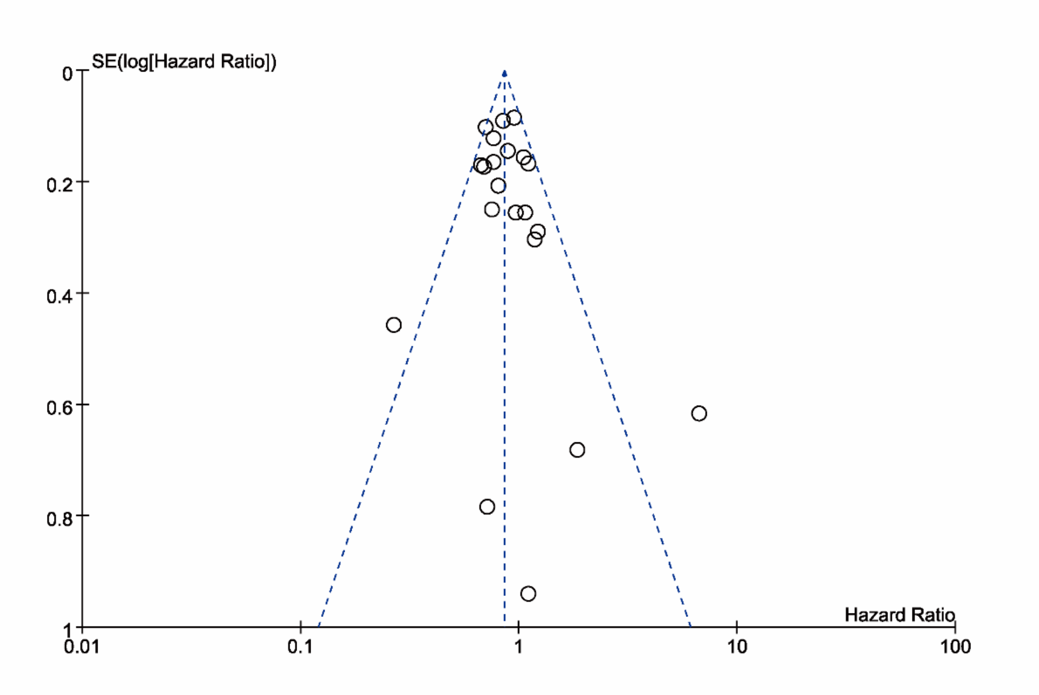

Supplementary Fig. 3 Hazard ratios and 95% confidence intervals of the comparison of overall survival between GBM patients with temozolomide and carmustine treatment. CI: confidence interval, df: degrees of freedom, SE: standard error

Study or Subgroup	log[Hazard Ratio]	SE	Weight	Hazard Ratio IV, Fixed, 95% CI
Illic, R 2017	-0.4463	0.2796	49.0%	0.64 [0.37, 1.11]
Vinjamuri, M 2009	-0.4308	0.2739	51.0%	0.65 [0.38, 1.11]
**Total (95% CI)**			**100.0%**	**0.65 [0.44, 0.95]**

Heterogeneity: Chi² = 0.00, df = 1 (P = 0.97); I² = 0%

Test for overall effect: Z = 2.24 (P = 0.03)

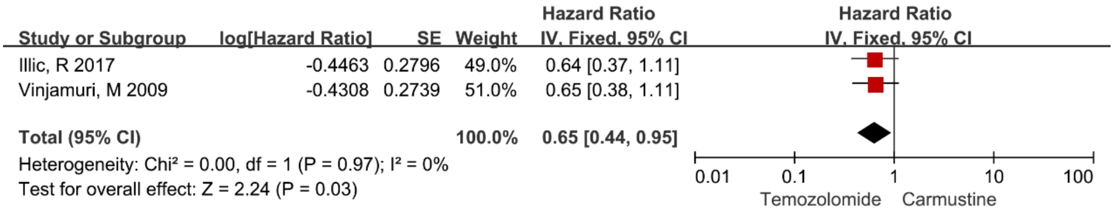

Supplement: Supplementary file 1 [file Data_Sheet_1.pdf]
